# miR-30 Regulates Mitochondrial Fission through Targeting p53 and the Dynamin-Related Protein-1 Pathway

**DOI:** 10.1371/journal.pgen.1000795

**Published:** 2010-01-08

**Authors:** Jincheng Li, Stefan Donath, Yanrui Li, Danian Qin, Bellur S. Prabhakar, Peifeng Li

**Affiliations:** 1 Department of Physiology, Shantou University School of Medicine, Shantou, China; 2 Franz-Volhard-Clinics, HELIOS Clinics GmBH, Berlin, Germany; 3 College of Medicine, University of Illinois at Chicago, Chicago, Illinois, United States of America; University of California San Francisco, United States of America

## Abstract

miRNAs participate in the regulation of apoptosis. However, it remains largely unknown as to how miRNAs are integrated into the apoptotic program. Mitochondrial fission is involved in the initiation of apoptosis. It is not yet clear whether miRNAs are able to regulate mitochondrial fission. Here we report that miR-30 family members are able to regulate apoptosis by targeting the mitochondrial fission machinery. Our data show that miR-30 family members can inhibit mitochondrial fission and the consequent apoptosis. In exploring the underlying molecular mechanism, we identified that miR-30 family members can suppress p53 expression. In response to the apoptotic stimulation, the expression levels of miR-30 family members were reduced, whereas p53 was upregulated. p53 transcriptionally activated the mitochondrial fission protein, dynamin-related protein-1 (Drp1). The latter conveyed the apoptotic signal of p53 by initiating the mitochondrial fission program. miR-30 family members inhibited mitochondrial fission through suppressing the expression of p53 and its downstream target Drp1. Our data reveal a novel model in which a miRNA can regulate apoptosis through targeting the mitochondrial fission machinery.

## Author Summary

Apoptosis is related to the pathogenesis of many diseases such as tumors and neurodegenerative and cardiovascular disorders. Apoptosis is controlled by a variety of genes, and among them a protein called “p53” has been taken as a “death star” and is involved in the initiation of apoptosis. The upstream and downstream molecules that control and convey p53 apoptotic function remain to be further identified. MicroRNAs (miRNAs) are a class of small non-coding RNAs that mediate post-transcriptional gene silencing. Mitochondrial fission participates in the initiation of apoptosis and requires the activation of a protein called dynamin-related protein-1 (Drp1). Our present work has revealed that miR-30 can target p53, thereby inhibiting p53 expression. Furthermore, our data show that p53 is able to induce mitochondrial fission by transcriptionally regulating Drp1. In addition, miR-30 controls Drp1 activity and the consequent apoptosis through p53. Our findings may warrant future studies to explore the therapeutic approaches for apoptosis-related diseases by targeting the miR-30-p53-Drp1 pathway.

## Introduction

MicroRNAs (miRNAs) are a class of small non-coding RNAs that mediate post-transcriptional gene silencing. Recently, the work on miRNAs renovates our understanding about the regulation of apoptosis. Differential miRNAs have distinct roles with either pro- or anti-apoptosis [1],[2]. For example, miR-1 participates in the initiation of apoptosis [3], whereas miR-21 is able to inhibit apoptosis [4],[5]. Although miRNAs are involved in apoptosis, it remains largely unknown as to how they are integrated into the apoptotic program.

Mitochondrial morphology is an important determinant of mitochondrial function [6]. Mitochondria constantly undergo fusion and fission that are necessary for the maintenance of organelle fidelity [7]. However, abnormal mitochondrial fusion and fission participate in the regulation of apoptosis. Mitochondrial fusion is able to inhibit apoptosis, while mitochondrial fission is involved in the initiation of apoptosis [8].

The heart is an organ composed of terminally differentiated cardiomyocytes. Since cardiomyocyte loss cannot be compensated by efficient cell proliferation, induction of apoptosis in cardiomyocytes may lead to the pathophysiological disorders. Apoptosis is related to cardiac diseases such as myocardial infarction, cardiomyopathy, cardiac hypertrophy and anthracycline-induced cardiotoxicity [9]–[15]. It is of note that cardiomyocytes are enriched in mitochondria. Hitherto, it remains largely unknown as to whether the abnormal mitochondrial fission plays a role in the initiation of cardiomyocyte apoptosis.

p53 can trigger cell death via apoptosis [16]–[18]. It initiates apoptosis through the intrinsic pathway by transcriptionally regulating the gene products of the proapoptotic proteins. For example, it can transactivate Bax, which translocates from the cytosol to mitochondria where it induces the release of apoptotic proteins [19]. p53 up-regulated modulator of apoptosis (PUMA) is rapidly induced by p53. It is exclusively located in mitochondria and can bind to Bcl-2 and Bcl-x(L), thereby inducing cytochrome c release and the consequent activation of caspase-9 and caspase-3 [20],[21]. Although several proapoptotic factors have been identified to be able to mediate the death signals of p53, the exact molecular mechanism by which p53 activates the intrinsic apoptotic pathway remains to be fully elucidated.

Mitochondrial fission requires the activation of a dynamin-related protein-1 (Drp1) [22]. Drp1 is a GTPase that causes scission of the mitochondrial outer membrane, resulting in fission of mitochondrial tubules into fragments. It is also responsible for cytochrome c release and caspase activation [7]. Although p53 is able to initiate apoptosis through the mitochondrial pathway, it is not yet clear whether Drp1 can be a downstream mediator of p53 in initiating the mitochondrial apoptotic pathway.

Our present work reveals that miR-30 family members are able to inhibit mitochondrial fission and apoptosis. We identified that p53 is a target of miR-30 family members. p53 induces mitochondrial fission by transcriptionally upregulating Drp1 expression. miR-30 can inhibit mitochondrial fission through suppressing the expression of p53 and its downstream target Drp1. Our data reveal a novel mitochondrial fission pathway composed of p53-Drp1 targeted by miR-30.

## Results

### Oxidative stress induces a decrease in miR-30 family members but an elevation in p53

**Figure 1:**
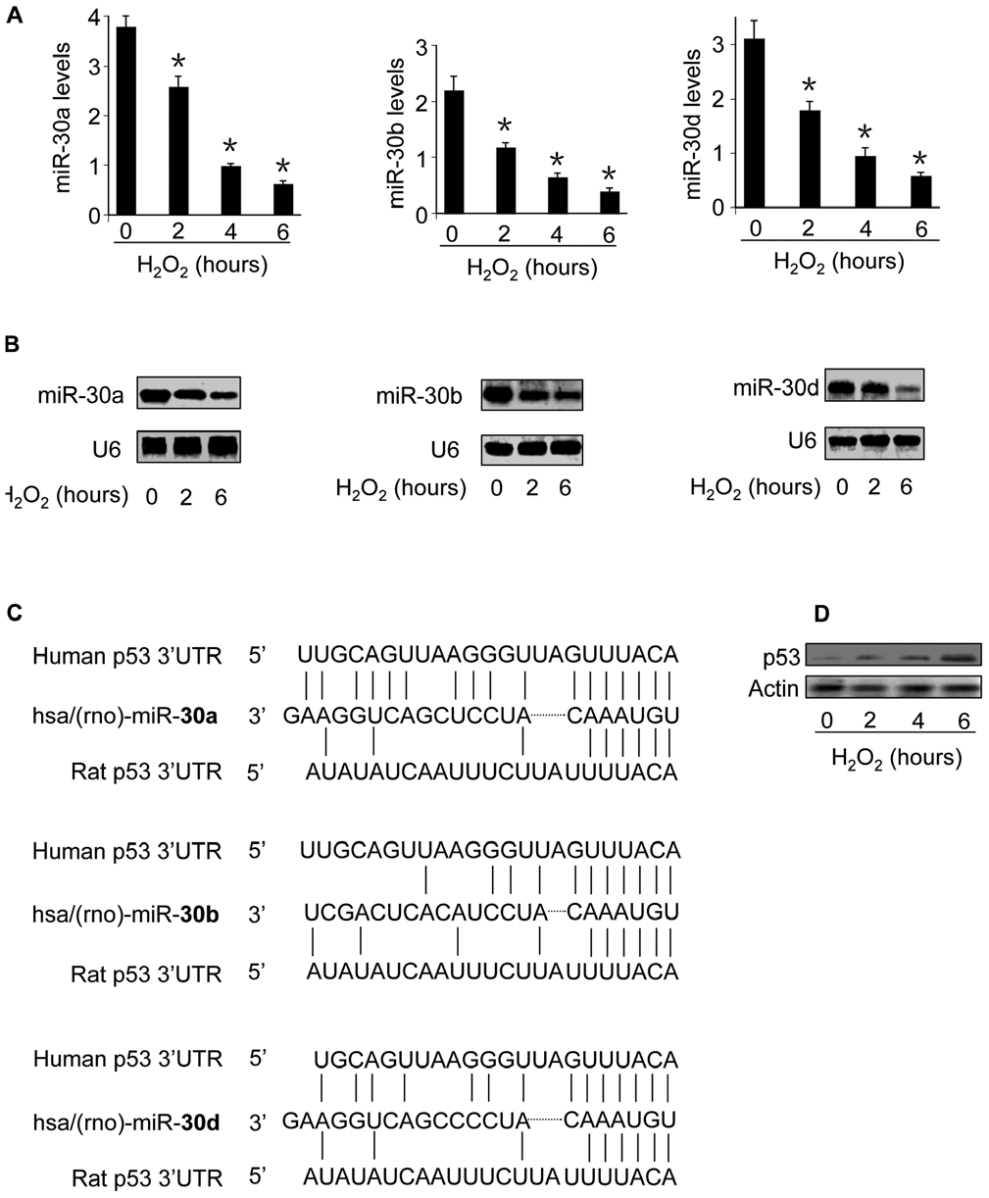
The levels of miR-30 family members are reduced whereas p53 is upregulated in response to oxidative stress. (A,B) miR-30a, miR-30b, and miR-30d levels are reduced. Cardiomyocytes were treated with 100 µM hydrogen peroxide, and harvested at the indicated time. miR-30a, miR-30b, and miR-30d levels were analyzed by qRT-PCR (A). *p<0.05, compared with the control. Their expression levels were detected by Northern blot (B). U6 was used as a loading control. (C) p53 is a potential target of miR-30a, miR-30b, and miR-30d. The miR-30 targeting sites in 3′UTRs of human and rat p53 are shown. (D) p53 is upregulated by oxidative stress. Cardiomyocytes were treated with as described for (A). p53 was analyzed by immunoblotting.

Oxidative stress has been well documented to be able to mediate the apoptotic signal, but the underlying molecular mechanism remains further elusive. miRNAs are involved in the control of apoptotic program. miR-30 family members include miR-30a, miR-30b, miR-30c, miR-30d and miR-30e. The hearts abundantly express miR-30 family members [23]–[25]. We analyzed their levels in response to oxidative stress stimulation. The quantitative real-time PCR and Northern blot analysis demonstrated that miR-30a, miR-30b and miR-30d levels were significantly reduced (Figure 1A and 1B). However, miR-30c and miR-30e levels were not significantly altered as revealed by quantitative real-time PCR (data not shown). We analyzed the expression levels of a control miRNA, miR-128, and observed that its levels were elevated in response to hydrogen peroxide treatment (Figure S1), suggesting that different miRNAs have a distinct response to oxidative stress. The alterations in miR-30 family members promoted us to search for their downstream targets. We analyzed the potential targets of miR-30 family members using the program of http://www.targetscan.org, and found that p53 is a potential target of miR-30a, miR-30b and miR-30d (Figure 1C). p53 also is the potential target of miR-30c and miR-30e (Figure S2A). Subsequently, we analyzed p53 expression levels and observed that it was upregulated in response to hydrogen peroxide treatment (Figure 1D). Thus, it appears that oxidative stress induces an inverse alteration in miR-30 family members and p53.

### miR-30 family members participate in the regulation of p53 expression

**Figure 2:**
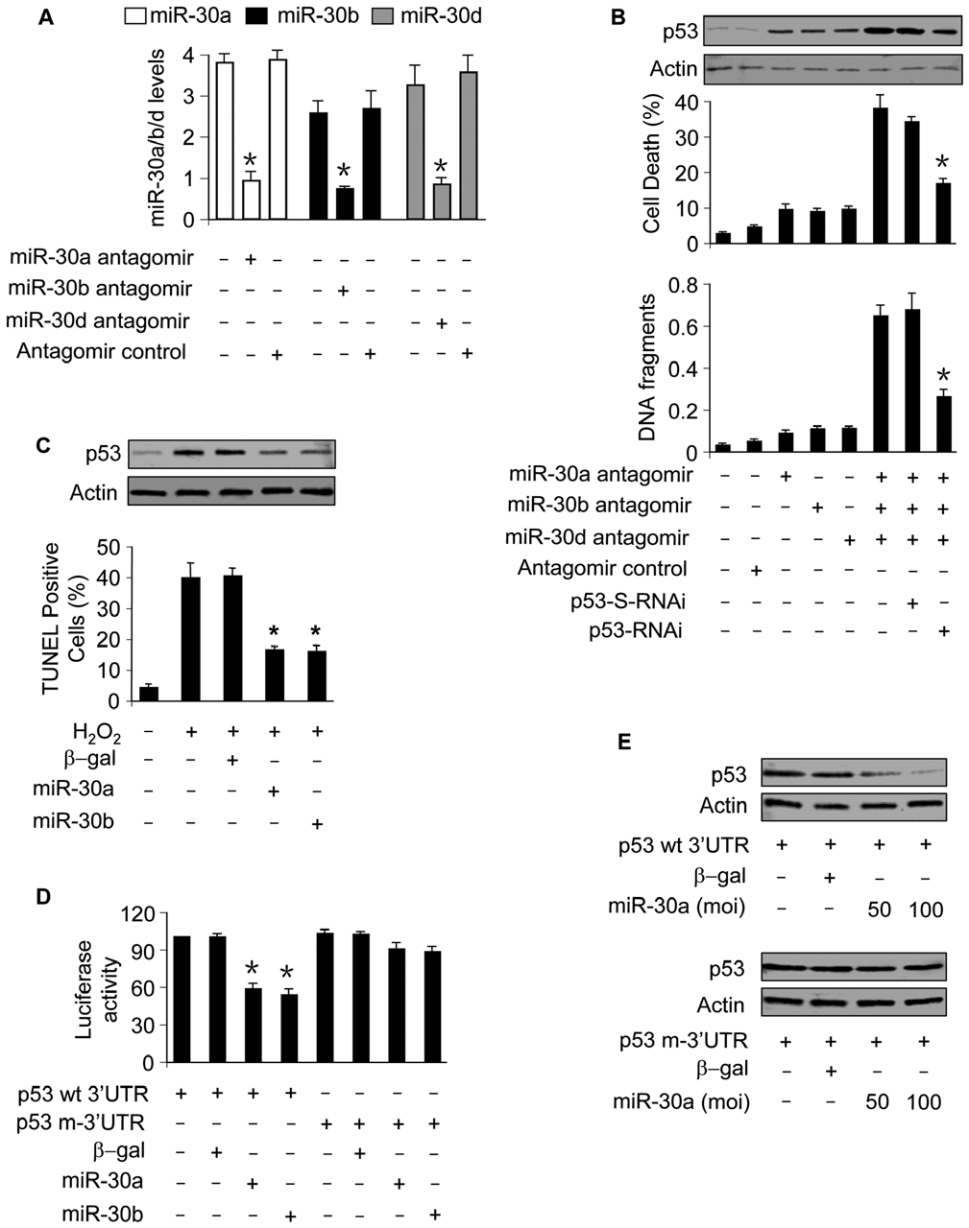
miR-30a, miR-30b, and miR-30d participate in the regulation of p53 expression. (A) Analysis of miR-30 levels. Cardiomyocytes were transfected with the antagomirs or the antagomir control at 100 nM, and harvested 48 h after transfection for the analysis of miR-30 levels. *p<0.05, compared with the control without treatment. (B) p53 is responsible for apoptosis induced by knockdown of miR-30. Cardiomyocytes were infected with Adp53RNAi or p53-S-RNAi at a moi of 50. 24 h after infection, cells were transfected with the antagomirs at 100 nM/each or the antagomir control at 300 nM. DNA fragmentation and p53 levels were analyzed 24 h after transfection. Cell death was analyzed 48 h after transfection. *p<0.05, compared with the simultaneous knockdown of miR-30a, miR-30b, and miR-30d. (C) Enforced expression of miR-30a or miR-30b is able to inhibit apoptosis induced by hydrogen peroxide. Cardiomyocytes were infected with the adenoviral miR-30a, miR-30b at a moi of 100. Adenoviral β-galactosidase (β-gal) served as a control. 24 h after infection, cells were treated with 100 µM hydrogen peroxide. Immunoblot analysis of p53 and TUNEL assay were performed 6 h and 12 h after treatment, respectively. *p<0.05, compared with the hydrogen peroxide alone. (D) miR-30a and miR-30b can suppress p53 translation. HEK293 cells were infected with the adenoviral miR-30a or miR-30b at a moi of 100. 24 h after infection, cells were transfected with p53 with wild-type 3′UTR (p53 wt-3′UTR) or mutated 3′UTR (p53 m-3′UTR). 24 h after transfection cells were harvested for the analysis of luciferase activity. *p<0.05, compared with p53 wt-3′UTR alone. (E) miR-30a significantly suppresses the expression of p53 with wild-type 3′UTR. HEK293 cells were infected with the adenoviral miR-30a at the indicated moi. 24 h after infection, cells were transfected with p53 with wild-type 3′UTR (p53 wt-3′UTR) or mutated 3′UTR (p53 m-3′UTR). p53 levels were analyzed by immunoblot. Data are expressed as the mean±SEM of three independent experiments.

miRNAs are able to negatively regulate the gene expression [26]–[28]. We explored whether p53 expression can be regulated by miR-30. To this end, we first tested whether knockdown of miR-30 can alter the expression of endogenous p53. miR-30a expression levels could be reduced by its antagomir. The expression levels of miR-30b and miR-30d also could be reduced by their antagomirs (Figure 2A). The single administration of miR-30a, miR-30b or miR-30d antagomir could slightly elevate p53 expression levels. However, the simultaneous administration of the three antagomirs led to a significant increase in p53 levels. Concomitantly, apoptosis could be observed. We further tested whether p53 is involved in the initiation of apoptosis upon miR-30 knockdown. p53 RNAi was able to attenuate apoptosis induced by knockdown of miR-30 (Figure 2B). Because p53 also is a potential target of miR-30c and miR-30e, we test whether knockdown of miR-30c and miR-30e can influence p53 expression levels. Immunoblot analysis revealed that p53 expression levels were elevated upon knockdown of miR-30c and miR-30e (Figure S2B). Apoptosis could be induced upon knockdown of miR-30c and miR-30e (Figure S2C).

We next tested whether the exogenous miR-30 members are able to influence p53 expression. Cells infected with adenoviral miR-30a and miR-30b showed an elevation in their expression levels (Figure S3). Enforced expression of miR-30a or miR-30b could attenuate p53 levels and apoptosis upon hydrogen peroxide treatment (Figure 2C).

To understand whether the regulation of miR-30 on p53 occurs through a direct or indirect manner, we tested whether miR-30 can influence the translation ability of p53. miR-30a and miR-30b could inhibit the translation ability of wild type but not the mutated p53 3′UTR (Figure 2D). Concomitantly, the expression of p53 with wild type but not the mutated 3′UTR could be significantly suppressed by miR-30a (Figure 2E) or miR-30b (data not shown). These results suggest that p53 is a direct target of miR-30.

### p53 is involved in mediating mitochondrial fission

**Figure 3:**
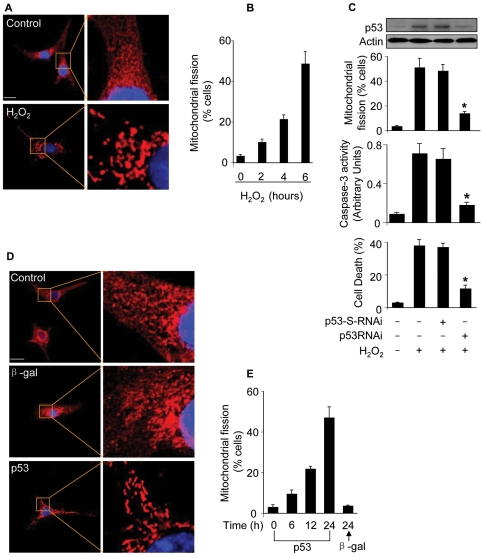
p53 induces mitochondrial fission. (A) The morphological alterations of mitochondria in cardiomyocytes upon treatment with hydrogen peroxide. In the control untreated cell, mitochondria demonstrate the characteristic reticular morphology. Upon hydrogen peroxide treatment, mitochondria disintegrate into numerous round fragments of varying size. Bar = 10 µm. (B) The percentage of cells undergoing mitochondrial fission. Cardiomyocytes were treated as described for (A), and harvested at the indicated time for detecting mitochondrial fission. (C) Knockdown of endogenous p53 attenuates mitochondrial fission. Cardiomyocytes were infected with Adp53RNAi or p53-S-RNAi at a moi of 50. 24 h after infection, cells were treated with hydrogen peroxide, and harvested 6 h after treatment for the detection of p53 protein levels and mitochondrial fission. Cell death and caspase-3 activity were analyzed 24 h after treatment. *p<0.05, compared with hydrogen peroxide alone. (D, E) p53 induces mitochondrial fission. Cardiomyocytes were infected with Adp53 or Adβ-gal at a moi of 50. Mitochondrial fission was analyzed by the morphology (D) or by counting the fission-positive cells (E). Data are expressed as the mean±SEM of three independent experiments.

We tested whether hydrogen peroxide can induce mitochondrial fission in cardiomyocytes. To this end, mitoTracker and mitochondria-targeted GFP were employed to monitor mitochondrial morphology. Mitochondria in the control untreated cells demonstrated reticular structure, in contrast, hydrogen peroxide treatment led to mitochondrial fragmentation as revealed by mitoTracker staining (Figure 3A). A similar pattern could be observed in cells monitored by mitochondria-targeted GFP (Figure S4). We counted the cells with mitochondrial fission. A time-dependent increase of cells with mitochondrial fission could be observed in response to hydrogen peroxide treatment (Figure 3B). To understand whether endogenous p53 participates in the induction of mitochondrial fission, we employed p53 RNAi to knockdown p53. Knockdown of p53 led to a reduction in mitochondrial fission, caspase-3 activation and death in cardiomyocytes upon hydrogen peroxide treatment (Figure 3C). Thus, it appears that endogenous p53 is involved in mediating mitochondrial fission induced by hydrogen peroxide.

Further, we detected whether exogenous p53 can induce mitochondrial fission. Enforced expression of p53 indeed led to the occurrence of mitochondrial fission as revealed by the morphology (Figure 3D) and the counting of cells with mitochondrial fission (Figure 3E). Taken together, it appears that p53 is able to induce mitochondrial fission.

### p53 is able to upregulate Drp1

**Figure 4:**
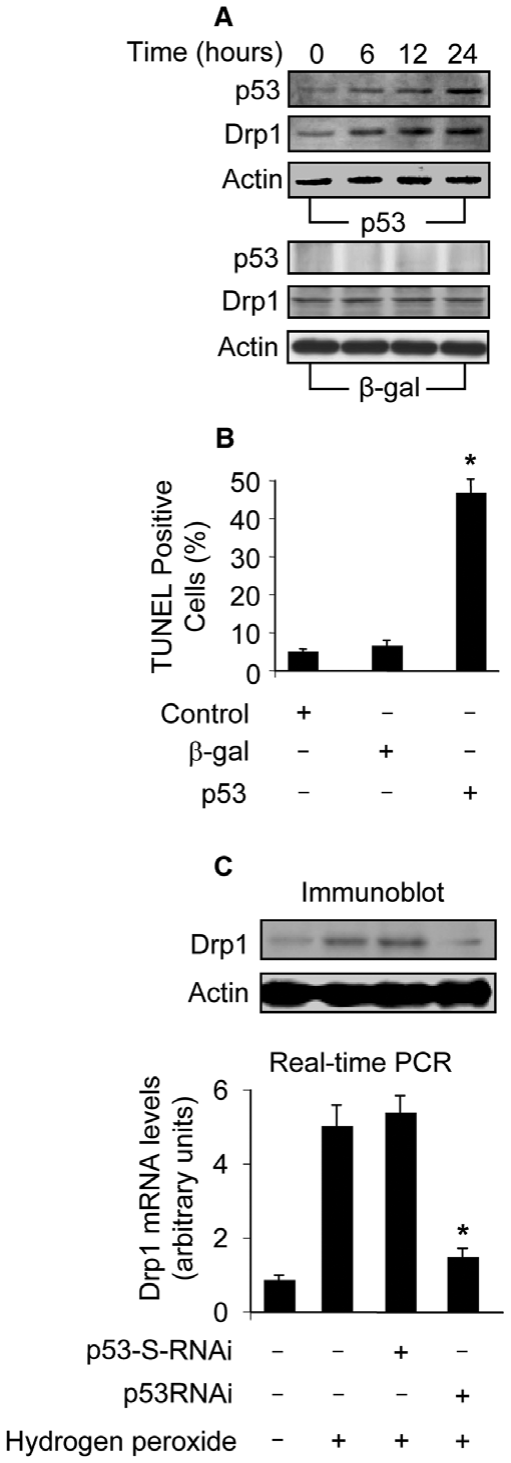
Drp1 is upregulated by p53. (A) p53 induces an elevated level of Drp1. Cardiomyocytes were infected with Adp53 or Adβ-gal at a moi of 50. p53 and Drp1 were analyzed by immunoblot. (B) p53 induces apoptosis. Cardiomyocytes were treated as described for (A). Apoptosis was analyzed by TUNEL assay. *p<0.05, compared with the control. (C) Knockdown of endogenous p53 attenuates Drp1 elevation induced by hydrogen peroxide. Cardiomyocytes were infected with Adp53RNAi or Adp53-S-RNAi at a moi of 50. 24 h after infection, cells were treated with hydrogen peroxide. Cells were harvested 6 h after treatment for the detection of Drp1 protein levels (upper panel) or mRNA levels using Real Time PCR (low panel). The levels of Drp1 mRNA analyzed by qRT-PCR were normalized to that of GAPDH. *p<0.05, compared with hydrogen peroxide alone. Data are expressed as the mean±SEM of three independent experiments.

Drp1 is an initiator of mitochondrial fission. The occurrence of mitochondrial fission led us to consider whether Drp1 expression levels were altered in response to p53 stimulation. Enforced expression of p53 led to an elevation in Drp1 protein levels (Figure 4A). Concomitantly, apoptosis could be detected in response to p53 stimulation (Figure 4B). We analyzed Drp1 expression levels upon hydrogen peroxide treatment, and Drp1 protein and mRNA levels were elevated. Such an elevation could be attenuated by knockdown of p53 (Figure 4C). These data suggest that p53 is able to upregulate Drp1 expression.

### Drp1 is a transcriptional target of p53

**Figure 5:**
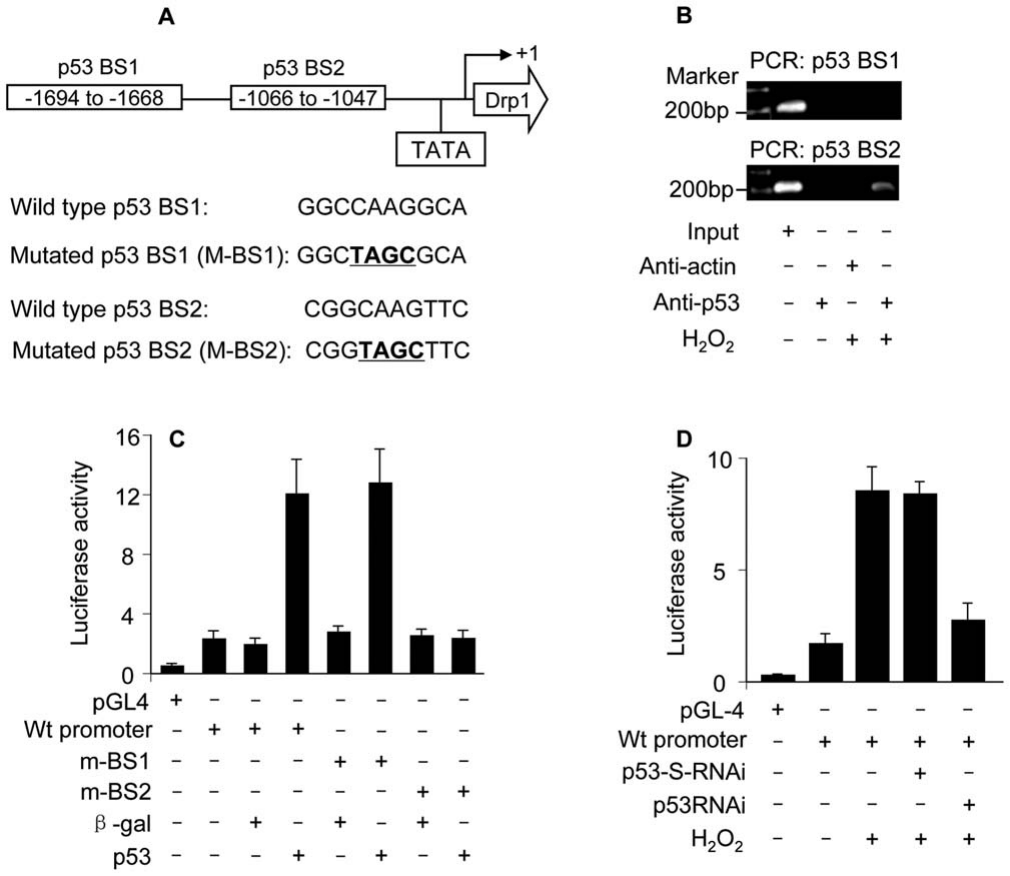
p53 binds to and activates Drp1 promoter. (A) The potential p53 binding sites in the Drp1 promoter region. Drp1 promoter region contains two potential p53 DNA binding sites (indicated as BS1 and BS2). The mutations were introduced to BS1 and BS2 (the converted nucleotides are underlined), respectively. (B) ChIP analysis of in vivo endogenous p53 binding to Drp1 promoter upon hydrogen peroxide treatment. 6 h after treatment cells were harvested for the ChIP analysis. Chromatin-bound DNA was immunoprecipitated with the anti-p53 antibody. Anti-actin antibody was used as a negative control. Immunoprecipitated DNA was analyzed by PCR using a primer combination that encompassed p53 BS1 (upper panel) or BS2 (lower panel). (C) p53 activates Drp1 promoter activity. Cardiomyocytes were infected with Adp53 or Adβ-gal at a moi of 50. 24 h after infection cells were transfected with the constructs of the empty vector (pGL-4), Drp1 wild-type promoter, or the mutated Drp1 promoters (m-BS1 and m-BS2), respectively. Firefly luciferase activities were normalized to Renilla luciferase activities. Data are expressed as the relative luciferase activity. (D) Hydrogen peroxide activates Drp1 promoter activity. Cardiomyocytes were infected with Adp53RNAi or Adp53-S-RNAi at a moi of 50. 24 h after infection cells were transfected with the constructs. 24 h after transfection cells were exposed to hydrogen peroxide. Data are expressed as the mean±SEM of three independent experiments.

The upregulation of Drp1 by p53 raises a question as to whether it occurs in a direct or indirect manner. According to the consensus binding site of p53, PuPuPuC(A/T)(T/A)GPyPyPy (N)_0–14_ PuPuPuC(A/T) (T/A)GPyPyPy, where N stands for any nucleotide [29], Drp1 promoter region contains two optimal p53 binding sites (p53 BS1, −1694 to −1668: GGCCAAGGCAAAGGAAAGTCAAGTAT; p53 BS2, −1066 to −1047: CGGCAAGTTCGTCTTGACT) (Figure 5A).

We initially tested whether endogenous p53 indeed can bind to the promoter region of Drp1. ChIP assay showed that p53 bound to Drp1 promoter region encompassing BS2 upon hydrogen peroxide treatment (the low panel in Figure 5B). No association was detectable in Drp1 promoter region encompassing BS1 upon hydrogen peroxide treatment (the upper panel in Figure 5B). These data suggest that BS2 in Drp1 promoter region appears to be the binding site of endogenous p53.

We next tested whether p53 can influence Drp1 promoter activity. The wild type Drp1 promoter activity was dramatically activated by enforced expression of p53 but not the negative control β-galactosidase. The introduction of mutations in p53 BS2 but not p53 BS1 resulted in the inability of p53 to activate Drp1 promoter activity, indicating that BS2 is the targeting site of p53 (Figure 5C).

Further, we tested whether endogenous p53 can influence Drp1 promoter activity. Hydrogen peroxide treatment led to an elevated Drp1 promoter activity. p53 RNAi could reduce the effect of hydrogen peroxide on Drp1 promoter activity (Figure 5D), suggesting that p53 is a mediator of hydrogen peroxide to activate Drp1 promoter. Taken together, it appears that Drp1 is a transcriptional target of p53.

### Drp1 is required for mitochondrial fission in p53-dependent apoptosis

**Figure 6:**
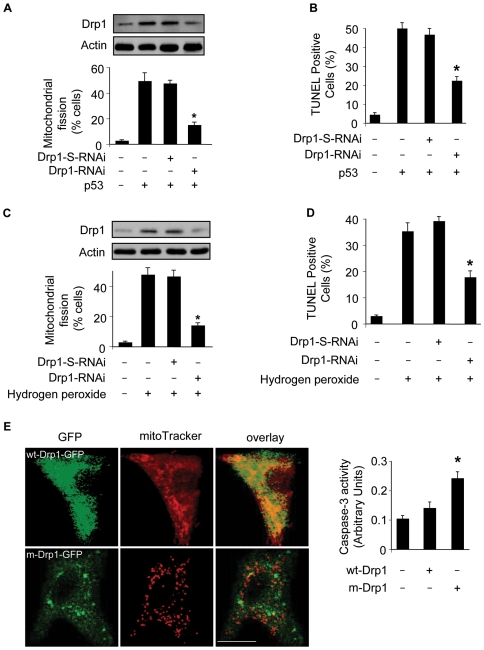
Drp1 is required for p53 to trigger mitochondrial fission. (A) Knockdown of Drp1 abolishes mitochondrial fission induced by p53. Cardiomyocytes were infected with AdDrp1RNAi or AdDrp1-S-RNAi at a moi of 70. 24 h after infection cells were treated with Adp53 at a moi of 50. Cells were harvested 24 h after treatment for the detection of Drp1 protein levels (upper panel) or mitochondrial fission (low panel). *p<0.05, compared with p53 alone. (B) Knockdown of Drp1 abolishes apoptosis induced by p53. Cardiomyocytes were treated as described for (A). TUNEL assay was performed 36 h after p53 infection. *p<0.05, compared with p53 alone. (C) Knockdown of Drp1 abolishes mitochondrial fission induced by hydrogen peroxide. Cardiomyocytes were treated with Drp1 RNAi as described for (A). Cells were harvested 6 h after treatment for the detection of Drp1 protein levels (upper panel) or mitochondrial fission (low panel). *p<0.05, compared with hydrogen peroxide alone. (D) Knockdown of Drp1 abolishes apoptosis induced by hydrogen peroxide. Cardiomyocytes were treated as described for (C). TUNEL assay was performed 12 h after treatment. *p<0.05, compared with hydrogen peroxide alone. (E) Overexpression of the mutated Drp1 induces mitochondrial fragmentation and caspase-3 activation. Cardiomyocytes were transfected with the constructs of wild-type Drp1-GFP (wt-Drp1-GFP) or the mutated Drp1-GFP (m-Drp1-GFP). Cells were stained with mitoTracker to monitor mitochondrial morphology. Caspase-3 activity was quantitatively analyzed (right panel). *p<0.05, compared with the control. Data are expressed as the mean±SEM of three independent experiments.

We tested whether Drp1 is involved in p53-induced mitochondrial fission. To this end, we employed RNAi technology to knockdown Drp1. Drp1 knockdown could lead to a decrease in mitochondrial fission (Figure 6A) and apoptosis (Figure 6B) induced by exogenous p53. We detected whether Drp1 is involved in mitochondrial fission induced by hydrogen peroxide. Knockdown of Drp1 led to a reduction in mitochondrial fission (Figure 6C) and apoptosis (Figure 6D) induced by hydrogen peroxide. These results suggest that Drp1 is necessary for the induction of mitochondrial fission in p53-dependent apoptosis.

To further confirm the role of Drp1 in mitochondrial fission in cardiomyocytes, we tested whether overexpression of Drp1 can influence mitochondria. Because Drp1 function is regulated by its phosphorylation status [30], we thus employed the wild type Drp1 and the mutated form which cannot be phosphorylated [30]. As shown in Figure 6E, the mutated form of Drp1 induced mitochondrial fragmentation, and an elevated level of caspase-3 activity. These data suggest that overexpression of Drp1 is able to induce mitochondrial fission and apoptosis in cardiomyocytes.

### miR-30 members regulate mitochondrial fission through p53

**Figure 7:**
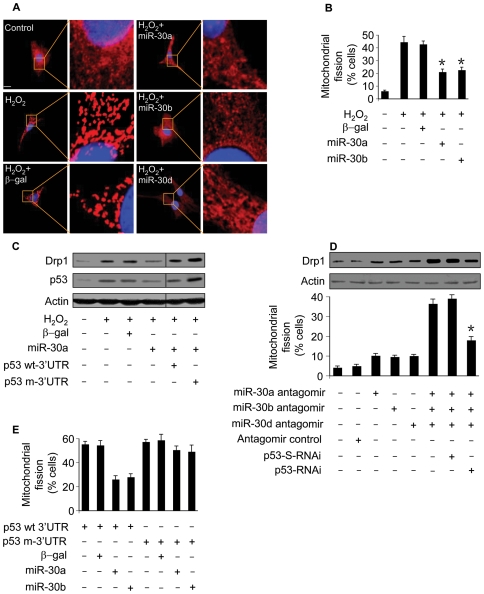
miR-30 family members regulate mitochondrial fission through p53. (A) miR-30 members inhibit mitochondrial fission. Cardiomyocytes were infected with adenoviral miR-30a, miR-30b, or miR-30d. 24 h after infection cells were treated with hydrogen peroxide. Mitochondrial fission was analyzed by the morphology. (B) The quantitative analysis of fission cells. Cardiomyocytes were treated as described for (A). The fission-positive cells were counted. *p<0.05, compared with hydrogen peroxide alone. (C) miR-30a attenuates the elevation in Drp1. Cells were coinfected with the adenoviral miR-30a and p53 with the wild-type 3′UTR (p53 wt3′UTR) or p53 with the mutated 3′UTR. 24 h after infection cells were treated with hydrogen peroxide, and harvested 6 h after treatment for the analysis of Drp1 and p53 levels by immunoblot. (D) Knockdown of miR-30 family members triggers mitochondrial fission. Cardiomyocytes were infected with Adp53RNAi or p53-S-RNAi at a moi of 50. 24 h after infection, cells were transfected with the antagomirs at 100 nM/each or the antagomir control at 300 nM. Mitochondrial fission and Drp1 were analyzed 24 h after transfection. *p<0.05, compared with the simultaneous knockdown of miR-30a, miR-30b, and miR-30d. (E) miR-30 members inhibit mitochondrial fission induced by p53. Cells were coinfected with the adenoviral miR-30a or miR-30b, along with p53 with the wild-type 3′UTR (p53 wt-3′UTR) or p53 with the mutated 3′UTR (p53 m-3′UTR). Mitochondrial fission was analyzed 24 h after infection.

To understand how miR-30 members are integrated into the apoptotic program, we tested whether miR-30 members can influence mitochondrial fission. Enforced expression of miR-30a, miR-30b and miR-30d could inhibit mitochondrial fission induced by hydrogen peroxide as revealed by the morphology (Figure 7A). Furthermore, the percentages of cells with mitochondrial fission were reduced upon overexpression of miR-30 members (7B). We attempted to understand whether miR-30a and miR-30b are able to affect Drp1. Drp1 expression was attenuated by enforced expression of miR-30a. To know if miR-30a exerts its effect on Drp1 through p53, we compared if p53 with wild type 3′UTR and p53 with the mutated 3′UTR have the same ability to regulate Drp1 in the presence of miR-30a. p53 with the mutated 3′UTR could be more effective than p53 with wild type 3′UTR to restore Drp1 levels in the presence of miR-30a (Figure 7C). A similar result was obtained in case of miR-30b (data not shown). We tested whether the endogenous miR-30 members participate in the regulation of Drp1 expression. The simultaneous knockdown of miR-30a, miR-30b and miR-30d resulted in an elevation in Drp1 levels and mitochondrial fission, and such elevation could be abolished by knockdown of p53 (Figure 7D).

To further understand the relationship between p53 and miR-30 in the mitochondrial fission machinery, we tested whether miR-30 members can influence exogenous p53 to induce mitochondrial fission. miR-30a or miR-30b could significantly attenuate mitochondrial fission induced by p53 with wild type 3′UTR but not p53 with mutated 3′UTR (Figure 7E). Our experiments used adenoviral β-galactosidase as a control for the adenoviral gene delivery. We further employed the miRNA Precursor-Negative Control to test whether the effect of miRNA is specific. Cells received the miR-30a precursor but not the Precursor-Negative Control could affect p53 expression levels, mitochondrial fission and apoptosis (Figure S5). Taken together, it appears that miR-30 members regulate mitochondrial fission through targeting p53.

### miR-30 members can influence mitochondrial fission and apoptosis in fibroblasts

**Figure 8:**
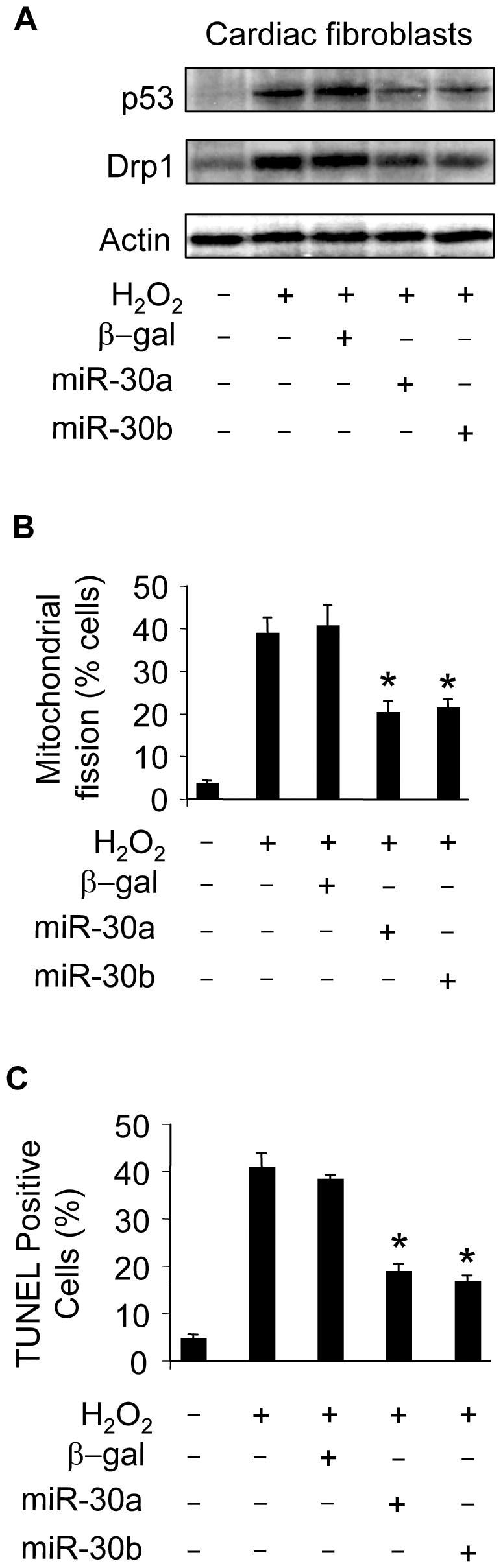
miR-30 can influence mitochondrial fission and apoptosis in cardiac fibroblasts. (A) Enforced expression of miR-30a or miR-30b is able to attenuate the expression levels of p53 and Drp1. Cardiomyocytes were infected with the adenoviral miR-30a or miR-30b at a moi of 100. Adenoviral β-galactosidase (β-gal) served as a control. 24 h after infection, cells were treated with 200 µM hydrogen peroxide. Immunoblot analysis of p53 and Drp1 were performed 6 h after treatment. (B,C) Enforced expression of miR-30a or miR-30b is able to inhibit mitochondrial fission and apoptosis induced by hydrogen peroxide. Cardiac fibroblasts were treated as described for (A). Mitochondrial fission (B) and apoptosis (C) were analyzed 6 h and 12 h after treatment, respectively. *p<0.05, compared with the hydrogen peroxide alone. Data are expressed as the mean±SEM of three independent experiments.

We tested whether miR-30 members can influence p53 and Drp1 expression, mitochondrial fission and apoptosis in cardiac fibroblasts. Enforced expression of miR-30a and miR-30b could inhibit p53 and Drp1 expression (Figure 8A). Mitochondrial fission (Figure 8B) and apoptosis (Figure 8C) were attenuated by enforced expression of miR-30a or miR-30b. These data suggest that miR-30 members can regulate mitochondrial fission and apoptosis in fibroblasts.

## Discussion

Our present work reveals that p53 can upregulate Drp1 expression in a transcription-dependent manner. Drp1 can convey the apoptotic signal of p53 by triggering mitochondrial fission. Thus, it appears that p53 and Drp1 constitute an axis in regulating mitochondrial fission and the consequent apoptosis. Strikingly, miR-30 members are integrated into apoptotic program by targeting this axis. Our data for the first time demonstrate that miRNAs are able to regulate mitochondrial fission machinery.

Drp1 has been shown to participate in initiating mitochondrial fission in apoptosis induced by a variety of stimuli. For example, staurosporin is able to trigger mitochondrial fission through Drp1 [31]. CD47 is an integrin-associated protein, and it induces apoptosis by causing Drp1 redistributions to mitochondria where it provokes the loss of mitochondrial membrane potential, production of reactive oxygen species and the disruption of the mitochondrial structure [32]. Nevertheless, the upstream signals that control the expression of Drp1 remain largely unknown. Our present work demonstrates that p53 is able to upregulate Drp1 expression at both mRNA and protein levels, and such upregulation occurs in a transcriptional manner. Thus, it appears that Drp1 expression is under the control of p53.

The patterns of mitochondrial fission in cardiomyocytes are in part different from that of other cell types. The heart function stringently depends on the ATP-generating pathway, and cardiomyocytes are therefore enriched in mitochondria. In particular, a characteristic of cardiomyocytes is that they are beating even in the culture conditions. These lines of evidence may provide an explanation for the features of mitochondrial fission in cardiomyocytes.

p53 is able to utilize the transcription-dependent pathway to initiate apoptosis. For example, p53 directly regulates the transcription of pro-apoptotic protein Bax [19], Bad [33] as well as PUMA [20],[21]. Furthermore, p53 can stimulate reactive oxygen species production [34],[35] and Fas/CD95 to redistribute to the cell surface [36]. In addition, p53 may induce cytochrome c release [37]. These observations indicate that p53 may use multiple mediators to convey its death signal. Our data that p53 requires Drp1 to initiate the mitochondrial apoptotic pathway shed new light on the understanding of the apoptotic machinery of p53.

miR-30 family members include miR-30a, -30b, -30c, -30d and -30e. They all have the same “seed sequence” in their 5′ terminuses, and are abundantly expressed in the heart under physiological condition. Nevertheless, their function in the heart, in particular, in apoptosis remains largely unknown. A most recent study shows that miR-30c regulates connective tissue growth factor in myocardial matrix remodelling [38]. Our present work shows that miR-30 family members including miR-30a, -30b and 30d are downregulated in response to apoptotic stimulation. Furthermore, they are able to suppress apoptosis. miRNAs have been shown to inhibit or promote apoptosis [39]–[41]. Hitherto, there has been no publication delineating the relationship between miRNAs and mitochondrial fission. Our present work provides novel evidence demonstrating that miRNAs are able to regulate apoptosis through affecting mitochondrial fission.

It is of note that upon stimulation with oxidative stress, miR-30 family members showed a differential response in which miR-30a, miR-30b and miR-30d were downregulated whereas miR-30c and miR-30e were not significantly altered. Although miR-30 family members have a similar sequence, they are expressed by genes localized in different chromosomes. It is possible that differential apoptotic stimuli may affect different genes expression. Reactive oxygen species such as hydrogen peroxide are widely involved in the pathogenesis of cardiac diseases, but a variety of other cardiac stressors also can induce apoptosis in cardiomyocytes. For example, hypoxia can induce apoptosis in cardiomyocytes [42]–[44]. Angiotensin II [11] and β-adrenergic agonists [45]–[47] are able to trigger apoptosis in cardiomyocytes. It would be interesting to study whether these apoptotic stimuli can induce apoptosis through targeting miR-30 family members.

p53 defects may lead to tumor pathogenesis in proliferating organs or tissues and thus, induction of apoptosis may be considered to be beneficial for the treatment of tumors that are related to abnormal cell proliferation. In contrast, the heart is an organ composed of differentiated postmitotic cardiac myocytes. Since the loss of cardiomyocytes cannot be compensated by efficient cell proliferation, the induction of apoptosis in cardiomyocytes may lead to pathophysiological disorders such as myocardial infarction and heart failure [48]–[54]. Therefore, p53 should be tightly controlled in the heart in order to prevent apoptosis in cardiomyocytes. It is of note that the human mir-30 target sites are only conserved among primates, and the sites in rat are non-canonical. Nevertheless, the hearts abundantly express miR-30 family members under physiological condition [23]–[25], and such an abundant expression pattern can provide one explanation for the low expression levels of p53 in the hearts. The regulation of miR-30 on p53 in mitochondrial fission machinery in other tissues and organs remains to be elucidated.

In summary, our finding that miR-30 family members can target p53-Drp1 axis thereby controlling mitochondrial fission and apoptosis may warrants future studies to explore the pathophysiological significance of regulating this pathway in cardiac diseases.

## Materials and Methods

### Cell cultures, treatment, and viability assay

Monolayer cultures of neonatal rat cardiac cells were isolated from 2-day-old Wistar rats and prepared as we described [55]. Briefly, after dissection hearts were washed, minced in HEPES-buffered saline solution contained 130 mM NaCl, 3 mM KCl, 1 mM NaH_2_PO_4_, 4 mM glucose and 20 mM HEPES (pH adjusted to 7.35 with NaOH). Tissues were then dispersed in a series of incubations at 37°C in HEPES-buffered saline solution containing 1.2 mg/ml pancreatin and 0.14 mg/ml collagenase (Worthington). After centrifugation cells were re-suspended in Dulbecco's modified Eagle medium/F-12 (GIBCO) containing 5% heat-inactivated horse serum, 0.1 mM ascorbate, insulin-transferring-sodium selenite media supplement, 100 U/ml penicillin, 100 µg/ml streptomycin, and 0.1 mM bromodeoxyuridine. The dissociated cells were pre-plated at 37°C for 1 h. Cardiac fibroblasts were cultured as we described [56]. In brief, in order to obtain cultures of cardiac fibroblasts, cells adherent to the culture dishes were cultured after preplating by adding the normal culture medium except that 10% fetal calf serum was added and bromodeoxyuridine was omitted. Cells used for experiment were at passage levels 6–8.

The treatment with hydrogen peroxide was performed as we described [57]. Cardiomyocytes were treated with 100 µM hydrogen peroxide, and cardiac fibroblasts were treated with 200 µM hydrogen peroxide. Cell death was determined by trypan blue exclusion, and the numbers of trypan Blue-positive and -negative cells were counted on a hemocytometer.

### Constructions of adenoviral rat Drp1 RNA interference (RNAi)

Drp1 RNAi sense sequence is 5′-CTCAGAGCAGTGGAAAGAG-3′; the antisense sequence is 5′-CTCTTTCCACTGCTCTGAG-3′. The scrambled Drp1 RNAi sense sequence is 5′-ATCGGATCAGGAACGGAAG-3′; the scrambled antisense sequence is 5′-CTTCCGTTCCTGATCCGAT-3′. They were cloned into pSilencer adeno 1.0-CMV vector (Ambion) according to the manufacturer's instructions.

### Constructions of rat Drp1 promoter and its mutated forms

Drp1 promoter was amplified from rat cardiomyocyte DNA using PCR. The forward primer is 5′-AAGGCTCAGAAAACATCACTG-3′. The reverse primer is 5′-GACTCCGGAAACACTGGACC-3′. The PCR fragment was cloned into the pcDNA 3.1 vector (Invitrogen), and then subcloned into the reporter plasmid, pGL4.17 (Promega). The introduction of mutations into the two putative p53 binding sites (BS1 wild type: 5′-GGCCAAGGCA-3′, mutant: 5′-GGCTAGCGCA-3′; BS2 wild type: 5′-CGGCAAGTTC-3′, mutant: 5′-CGGTAGCTTC-3′) was generated using QuikChange II XL Site-Directed Mutagenesis Kit (Stratagene) according to the manufacturer's instructions. All constructs were sequenced to check that only the desired mutations had been introduced.

### Adenoviral construction of miR-30a, miR-30b, miR-30d, and p53 with 3′UTR

miR-30a, miR-30b and miR-30d were synthesized by polymerase chain reaction using rat cardiomyocyte DNA as the template. For miR-30a, the upstream primer was 5′-CTAGGGCATATCTGAACGAGG-3′; the downstream primer was 5′-CACAGGAGGAACATTTCTGTG-3′. For miR-30b, the upstream primer was 5′-GGATACTCCAAGACAGCTGAC-3′; the downstream primer was 5′-TGTTTCTGTGGATTGTACACC-3′. For miR-30d, the upstream primer was 5′- CTGCCAGCAGAAGCAAGCAG-3′; the downstream primer was 5′- GTTGTCATTCAGCTCTGCAG-3′. p53 with 3′UTR was synthesized using reverse transcription polymerase chain reaction as we described [58]. The upstream primer was 5′-CCTGAAGACTGGATAACTGTC-3′. The downstream primer was 5′-CAAGTGGTAACAAAAGTTTATTG-3′. To produce p53 with mutated 3′UTR, the mutations (wild type 3′UTR: 5′-ATTTTACA-3′, mutated 3′UTR: 5′-ATTTACAC-3′) was generated using QuikChange II XL Site-Directed Mutagenesis Kit (Stratagene). The constructs were sequence verified. They were cloned into the Adeno-X™ Expression System (Clontech) according to the manufacturer's instructions. Adenoviral p53 without 3′UTR, adenovirus β-galactosidase (Adβ-gal), adenoviruses harboring p53 RNA interference (Adp53RNAi) and its scrambled form (Adp53-S-RNAi) were as we and other described [57],[59]. Viruses were amplified in 293 cells and purified on a CsCl gradient. Cells were infected with the virus at the indicated multiplicity of infection (moi). After washing with PBS, culture medium was added and cells were cultured until the indicated time.

### Preparation of the luciferase construct of p53 3′UTR

p53 wild type and mutated 3′UTRs were subcloned into the pGL3 vector (Promega) immediately downstream of the stop codon of the luciferase gene.

### Immunoblot analysis

Immunoblotting was carried out as we previously described [34]. Cells were lysed for 1 h at 4°C in a lysis buffer (20 mM Tris pH 7.5, 2 mM EDTA, 3 mM EGTA, 2 mM dithiothreitol (DTT), 250 mM sucrose, 0.1 mM phenylmethylsulfonyl fluoride, 1% Triton X-100) containing a protease inhibitor cocktail. Samples were subjected to 12% SDS-PAGE and transferred to nitrocellulose membranes. Equal protein loading was controlled by Ponceau Red staining of membranes. Blots were probed using the primary antibodies. The anti-Drp1 antibody was from Santa Cruz Biotechnology. The anti-p53 antibody was from Calbiochem. After four times washing with PBS Tween-20, the horseradish peroxidase-conjugated secondary antibodies were added. Antigen-antibody complexes were visualized by enhanced chemiluminescence.

### Chromatin immunoprecipitation (ChIP) analysis

ChIP was performed as we and other described [57],[60]. In brief, cells (0.2×10^8^) were washed with PBS and incubated for 10 min with 1% formaldehyde at room temperature. The cross-linking was quenched with 0.1 M glycine for 5 min. Cells were washed twice with PBS and lysed for 1 h at 4°C in a lysis buffer. The cell lysates were sonicated into chromatin fragments with an average length of 500 to 800 bp as assessed by agarose gel electrophoresis. The samples were precleared with Protein-A agarose (Roche) for 1 h at 4°C on a rocking platform, and 10 µg specific antibodies were added and rocked for overnight at 4°C. Immunoprecipitates were captured with 10% (vol/vol) Protein-A agarose for 4 h. Before use, Protein-A agarose was blocked twice at 4°C with salmon sperm DNA (1 µg/ml) that had been sheared to a 500-bp length and BSA (1 µg/ml) overnight. PCRs were performed with the primers that encompass p53 BS1 or BS2 of the rat Drp1 promoter. The oligonucleotides were as follows: BS1 (corresponding to a 225 bp fragment), Forward: 5′-GGCTGTATGTGTTCCATTAC-3′; reverse: 5′-AGACAGAAGAGAGTAGGCTC-3′. BS2 (corresponding to a 220 bp fragment), Forward: 5′-AGTAAAGCCTGTCTTGTGTG-3′; reverse: 5′-AAATAATCACAATATACTG-3′.

### Luciferase assay

For Drp1 promoter luciferase assay, cells were seeded in 6-well plates (2×10^5^ cells/well). They were transfected with the plasmid constructs using the Effectene Transfection Kit (Qiagen). Each well contains 0.6 µg luciferase reporter plasmids, 10 ng SV-Renilla luciferase plasmids as the internal control. Cells were harvested at the indicated time after transfection for the detection of luciferase activity using the Dual Luciferase Reporter Assay kit (Promega) according to the manufacturer's instructions. 20 µl of protein extracts were analyzed in a luminometer. Firefly luciferase activities were normalized to Renilla luciferase activity. For p53 3′UTR luciferase assay, cells were infected with the adenoviral miR-30a or miR-30b at a moi of 100. 24 h after infection, cells were transfected with the luciferase constructs of p53 with wild type 3′UTR or mutated 3′UTR.

### Quantitative real-time PCR

For quantitative real-time PCR (qRT-PCR), RNA was prepared by TRIzol reagent (Invitrogen). Total RNA was processed to cDNA by reverse transcription using the High Capacity cDNA Reverse Transcription Kit (Applied Biosystems). Real-time PCR using Power SYBR Green PCR Master Mix (Applied Biosystems) was carried out in triplicate in a 7500 Fast Real-Time PCR System (Applied Biosystems) according to the manufacturer's instructions. The sequences of Drp1 primers were Forward: 5′-AGCTGCAAGACGTCTTCAAC-3′; Reverse: 5′-CATTCTTCTGCTTCAACTCC-3′. Analysis was performed using the software supplied with the instrument. GAPDH forward primer: 5′-TGTTCCAGTATGACTCTACC-3′; Reverse: 5′-TGGGTTTCCCGTTGATGACC-3′. The levels of Drp1 mRNA analyzed by qRT-PCR were normalized to that of GAPDH. The specificity of the PCR amplification was confirmed by agarose gel electrophoresis.

The TaqMan MicroRNA Assays kits were employed to analyze the levels of miR-30 family members and miR-128 according to the manufacturer's instructions (Applied Biosystems). The levels of miR-30 family members and miR-128 analyzed by qRT-PCR were normalized to that of U6.

### Transfection of antagomir, miRNA precursor, or Drp1

The antagomirs designed to inhibit the expression of endogenous miR-30 members, and antimiR Negative Control 1 were obtained from Ambion. miR-30a precursor, and miRNA Precursor Molecules—Negative Control #1 were obtained from Ambion. The cDNA constructs of wild type Drp1-GFP and Drp1 mutant in which serine 656 was mutated to an alanine residue were kindly provided by Dr. Stefan Strack [30]. Cells were transfected using Lipofectamine 2000 (Invitrogen) according to the manufacturer's instruction.

### Northern blot analysis

Northern blot was performed as we and other described [57],[61],[62]. In brief, 40 µg RNA was run on a 15% polyacrylamide-urea gel, transferred to a Hybond N^+^ membrane (Amersham Bioscience). Membranes were hybridized with 10 pmol probes. The probes were produced by Integrated DNA Technologies. They were 5′-biotin labeled, and Locked Nucleic Acids (LNA) were used. Probes sequences for miR-30a: 5-CTTCCAGTCGAGGATGTTTACA-3′; miR-30b: 5′-AGCTGAGTGTAGGATGTTTACA-3′; miR-30d: 5′-CTTCCAGTCGGGGATGTTTACA-3′. U6 was used as a loading control, and its sequence was 5′-CACGAATTTGCGTGTCATCCTT-3′.

### Mitochondrial staining or fluorescent labeling

Mitochondrial staining was carried as we and others described with modifications [22],[63]. Briefly, cells were plated onto the cover-slips coated with 0.01% poly-L-lysine. After treatment they were stained for 20 min with 0.02 µM MitoTracker Red CMXRos (Molecular Probes). For fluorescent labeling of mitochondria, cells were transfected with pAcGFP1-mito which encodes a fusion of a mitochondrial targeting sequence derived from the precursor of subunit VIII of cytochrome c oxidase and the green fluorescent protein (Clontech). Mitochondria were imaged using a laser scanning confocal microscope (Zeiss LSM510 META).

### TUNEL assay

Apoptosis was assessed by terminal deoxynucleotidyl transferase-mediated dUTP nick-end-labeling (TUNEL) assay using a kit from Clontech following the kit's instructions. 150–200 cells were counted in 20–30 random fields.

### Detection of caspase-3 activity

Caspase-3 activity was detected using an assay kit (R&D System). The assay procedures were followed according to the kit instructions.

### Statistical analysis

The results are expressed as means±SEM. Data were evaluated by Student's t-test. A value of p<0.05 was considered significant.

## Supporting Information

Figure S1 miR-128 levels were increased upon treatment with hydrogen peroxide. Cardiomyocytes were treated with 100 µM hydrogen peroxide, and harvested at the indicated time for the detection of miR-128. *p<0.05 vs control. Data are expressed as the mean±SEM.
(0.07 MB TIF)

Figure S2 Knockdown of miR-30c and miR-30e leads to p53 upregulation and apoptosis. (A) p53 is a potential target of miR-30c and miR-30e. The targeting sites of miR-30c and miR-30e in 3′UTRs of human and rat p53 are shown. (B,C) Knockdown of miR-30c and miR-30e leads to p53 upregulation and apoptosis. Cardiomyocytes were transfected with the antagomirs of miR-30c or miR-30e at 100 nM or the antagomir control at 200 nM. p53 levels were analyzed 24 h after transfection (B). Apoptosis was analyzed by TUNEL assay 36 h after transfection (C). *p<0.05, compared with the control.
(0.17 MB TIF)

Figure S3 Analysis of miR-30a and miR-30b levels. Cardiomyocytes were infected with the adenoviral miR-30a, miR-30b, at a moi of 100. Adenoviral β-galactosidase (β-gal) served as a control. 24 h after infection, cells were treated with 100 µM hydrogen peroxide. miR-30a and miR-30b were analyzed 6 h after treatment. miR-30a and miR-30b levels in cells without hydrogen peroxide treatment were analyzed at the identical time.
(0.11 MB TIF)

Figure S4 Mitochondrial fission monitored by fluorescent labelling. Cardiomyocytes were transfected with the plasmid construct of pAcGFP1-mito encoding a mitochondria-targeted GFP, and then treated with 100 µM hydrogen peroxide. Mitochondrial morphology was monitored. Bar = 10 µm.
(0.94 MB TIF)

Figure S5 miR-30a precursor but not the Precursor-Negative Control could affect p53 expression levels, mitochondrial fission and apoptosis. Cardiomyocytes were transfected with the miR-30a precursor or the Precursor-Negative Control. 12 h after transfection, cells were treated with 100 µM hydrogen peroxide. p53 expression levels were analyzed by immunoblot (upper panel). The percentages of cells with mitochondrial fission and apoptosis are shown in the middle and low panels, respectively.
(0.16 MB TIF)
