# Correction: miR-30 Regulates Mitochondrial Fission through Targeting p53 and the Dynamin-Related Protein-1 Pathway

**DOI:** 10.1371/annotation/4050116d-8daa-4b5a-99e9-34cdd13f6a26

**Published:** 2010-01-21

**Authors:** Jincheng Li, Stefan Donath, Yanrui Li, Danian Qin, Bellur S. Prabhakar, Peifeng Li

Yanrui Li is incorrectly listed as a corresponding author for this manuscript. Instead, Yanrui Li should be listed as having contributed equally with Jincheng Li and Stefan Donath.

