# Supplementary figures and images for: miR-30 Regulates Mitochondrial Fission through Targeting p53 and the Dynamin-Related Protein-1 Pathway
Source: PLoS Genet. 2010 Jan 8;6(1):e1000795. doi: 10.1371/journal.pgen.1000795 (PMC2793031; doi:10.1371/journal.pgen.1000795)

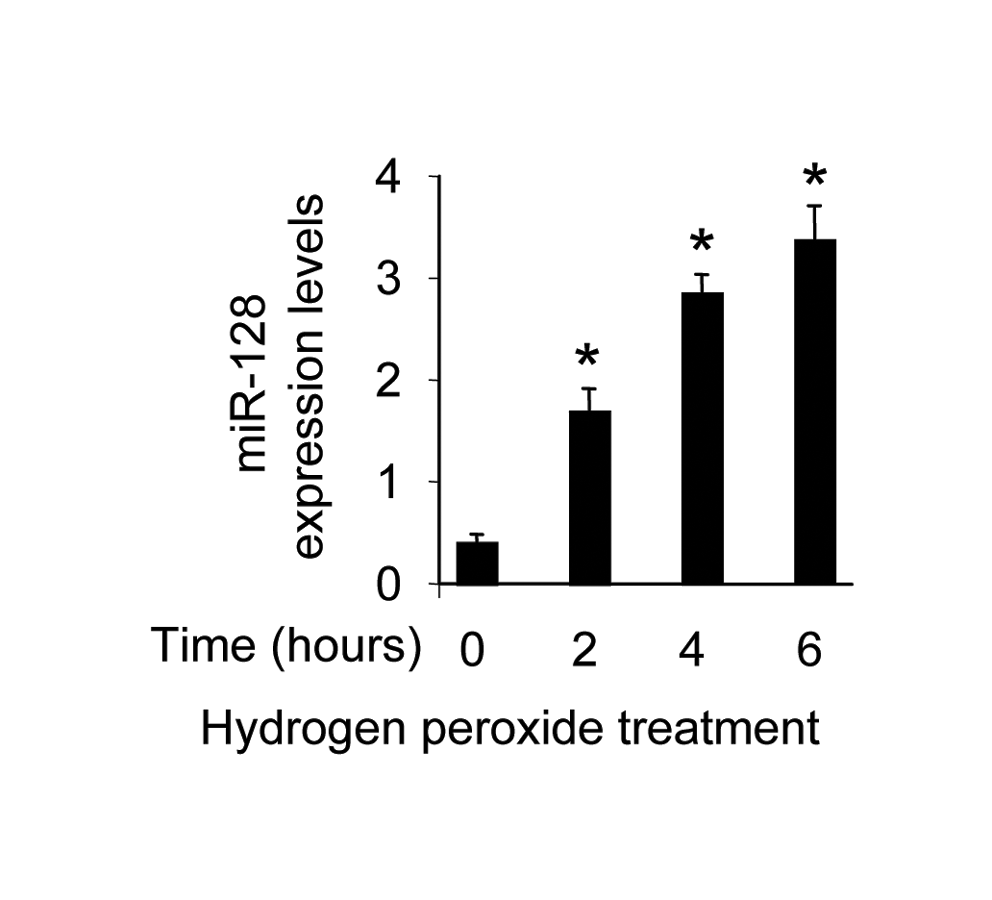

Supplement: Figure S1 — miR-128 levels were increased upon treatment with hydrogen peroxide. Cardiomyocytes were treated with 100 µM hydrogen peroxide, and harvested at the indicated time for the detection of miR-128. *p<0.05 vs control. Data are expressed as the mean±SEM. (0.07 MB TIF) [file pgen.1000795.s001.tif]

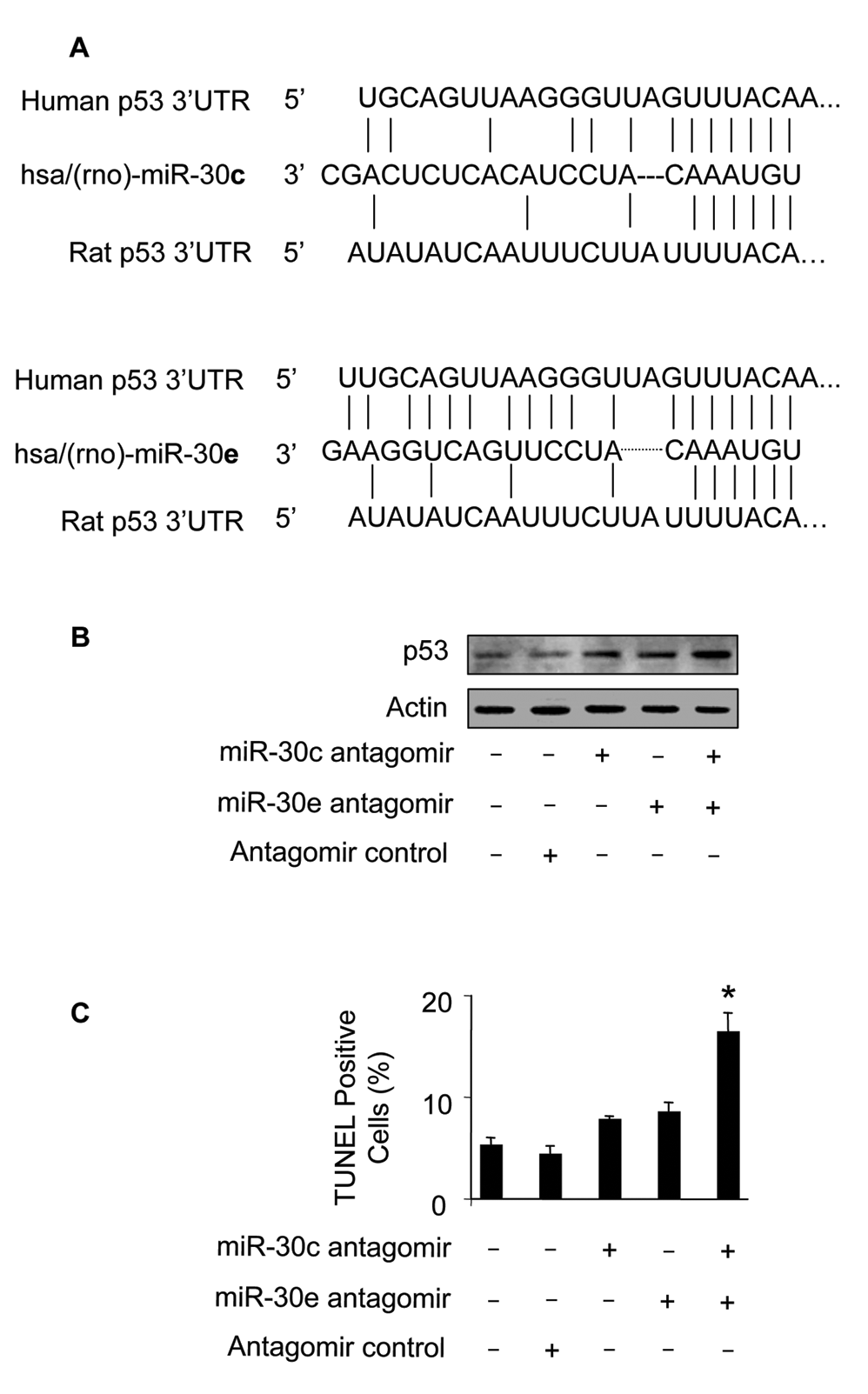

Supplement: Figure S2 — Knockdown of miR-30c and miR-30e leads to p53 upregulation and apoptosis. (A) p53 is a potential target of miR-30c and miR-30e. The targeting sites of miR-30c and miR-30e in 3′UTRs of human and rat p53 are shown. (B,C) Knockdown of miR-30c and miR-30e leads to p53 upregulation and apoptosis. Cardiomyocytes were transfected with the antagomirs of miR-30c or miR-30e at 100 nM or the antagomir control at 200 nM. p53 levels were analyzed 24 h after transfection (B). Apoptosis was analyzed by TUNEL assay 36 h after transfection (C). *p<0.05, compared with the control. (0.17 MB TIF) [file pgen.1000795.s002.tif]

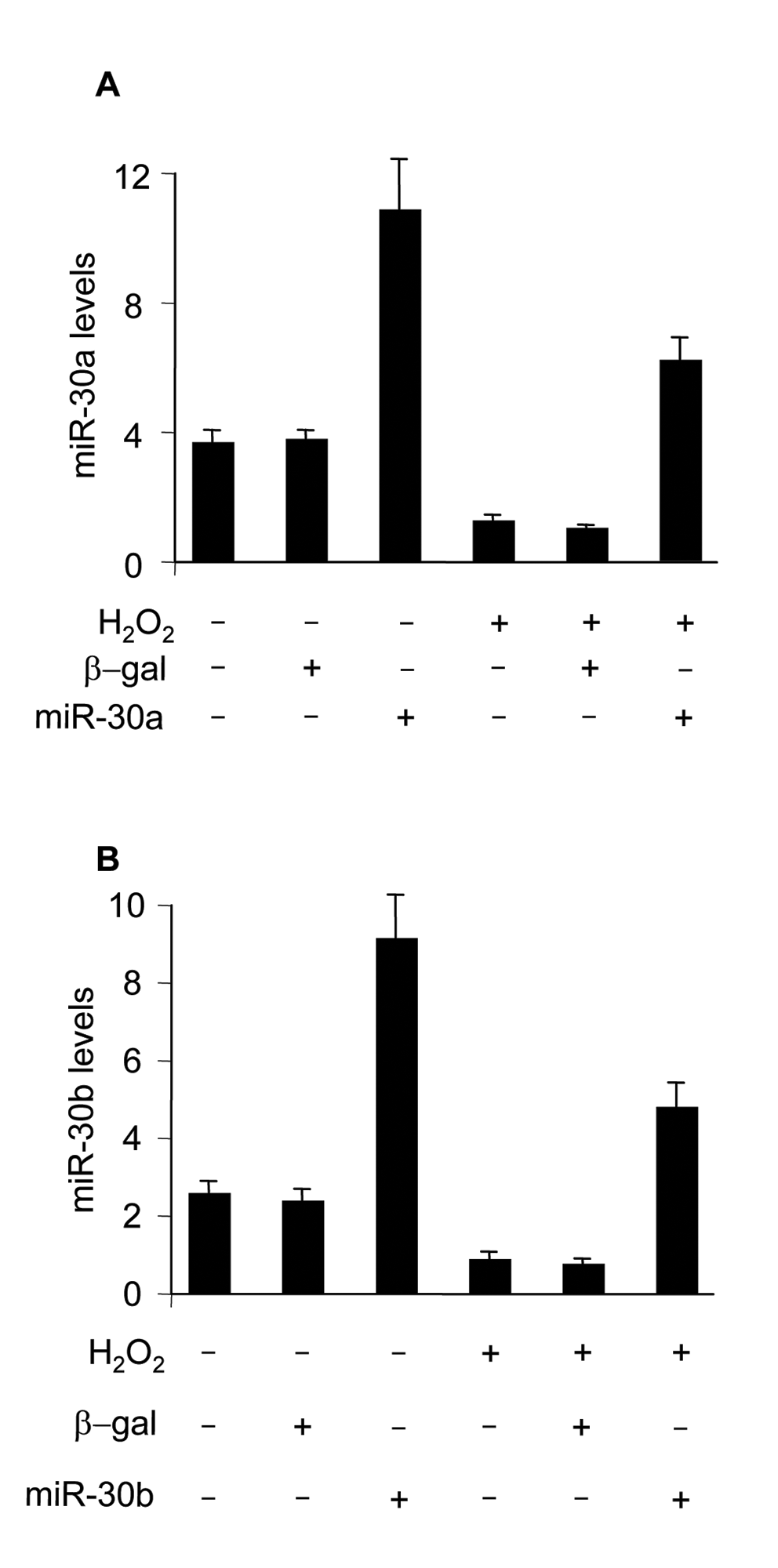

Supplement: Figure S3 — Analysis of miR-30a and miR-30b levels. Cardiomyocytes were infected with the adenoviral miR-30a, miR-30b, at a moi of 100. Adenoviral β-galactosidase (β-gal) served as a control. 24 h after infection, cells were treated with 100 µM hydrogen peroxide. miR-30a and miR-30b were analyzed 6 h after treatment. miR-30a and miR-30b levels in cells without hydrogen peroxide treatment were analyzed at the identical time. (0.11 MB TIF) [file pgen.1000795.s003.tif]

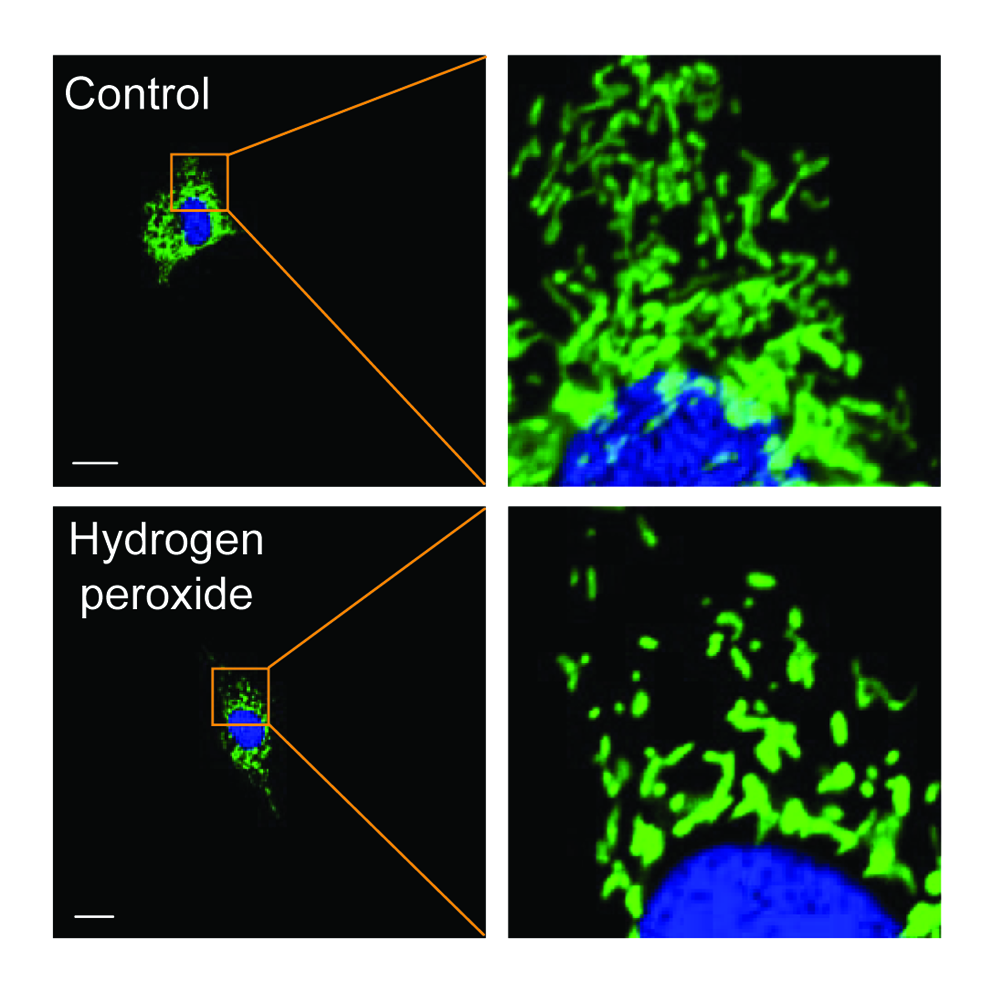

Supplement: Figure S4 — Mitochondrial fission monitored by fluorescent labelling. Cardiomyocytes were transfected with the plasmid construct of pAcGFP1-mito encoding a mitochondria-targeted GFP, and then treated with 100 µM hydrogen peroxide. Mitochondrial morphology was monitored. Bar = 10 µm. (0.94 MB TIF) [file pgen.1000795.s004.tif]

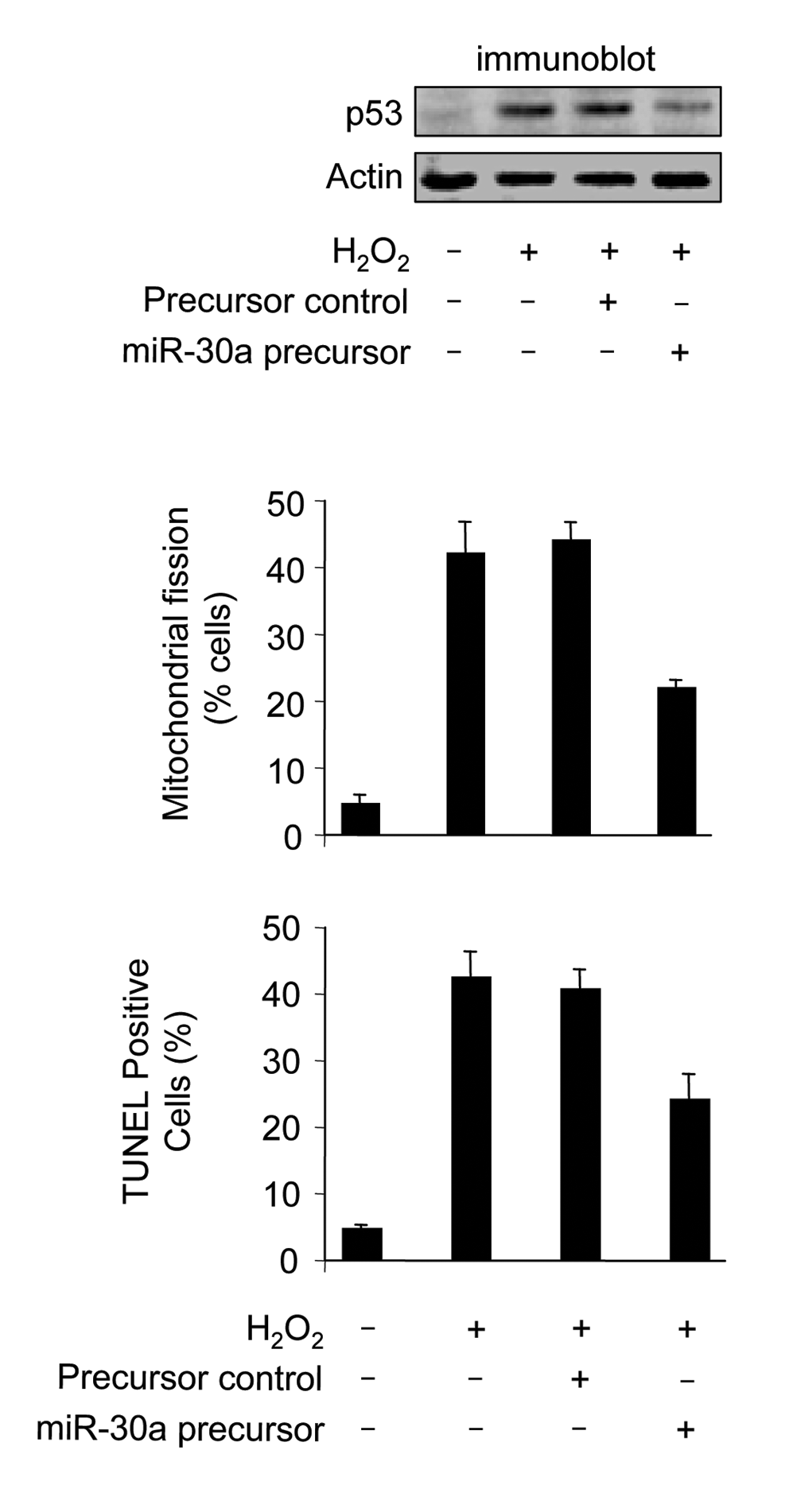

Supplement: Figure S5 — miR-30a precursor but not the Precursor-Negative Control could affect p53 expression levels, mitochondrial fission and apoptosis. Cardiomyocytes were transfected with the miR-30a precursor or the Precursor-Negative Control. 12 h after transfection, cells were treated with 100 µM hydrogen peroxide. p53 expression levels were analyzed by immunoblot (upper panel). The percentages of cells with mitochondrial fission and apoptosis are shown in the middle and low panels, respectively. (0.16 MB TIF) [file pgen.1000795.s005.tif]
